# Editorial: Language acquisition in diverse linguistic, social and cognitive circumstances, volume II

**DOI:** 10.3389/fpsyg.2023.1282163

**Published:** 2023-09-13

**Authors:** Maria Garraffa, Maria Teresa Guasti, Theodoros Marinis, Gary Morgan

**Affiliations:** ^1^Faculty of Medicine and Health Sciences, School of Health Sciences, University of East Anglia, Norwich, United Kingdom; ^2^Department of Psychology, University of Milano-Bicocca, Milan, Italy; ^3^Department of Linguistics, University of Konstanz, Konstanz, Germany; ^4^School of Health and Psychological Sciences, City University of London, London, United Kingdom

**Keywords:** language acquisition, attention deficit and hyperactivity disorder (ADHD), multilingualism, pragmatics, narrative abilities, developmental language disorder (DLD)

This volume is the second of the Research Topic “Language acquisition in diverse linguistic, social and cognitive circumstances,” (Volume 1—Garraffa et al., 2018). The new volume presents research on multilingual speakers, including underrepresented languages in a set of different linguistics abilities in both typical and atypical populations. It presents nine contributions, divided into two sections: one section on language competence in children with neurodevelopmental disorders and a second one on language learning in a multilingual context.

## Section 1: language competence in children with neurodevelopmental disorders

Studies on child language acquisition requires evidence that is representative of the typological language diversity present in the ~7,000 or so languages spoken in the world. This is often not the case in research: a recent review of language acquisition studies found that we have research evidence on around 103 languages, representing ~1.5% of the world's languages (Kidd and Garcia, 2022). More investigations in underrepresented languages are of particular importance in atypical language development, where data are scarce and there is a lack of resources for research. The relevance of testing underrepresented languages for atypical language development was addressed in Abu Bakar et al. on comprehension and production of Wh-sentences in children's speakers of Malay with and without developmental language disorder (DLD). The Malay speakers with DLD performed similarly to younger children with typical language development (TLD) and their language use reflected colloquial Malay with selective omissions of particles unlike age-matched children with TLD. Specific grammatical strategies are adopted by children with DLD in line with an immature acquisition of syntax, opening the debate of the need of qualitative and theoretical driven analyses of the production in children with DLD.

Research on bilingual speakers with a neurodevelopmental disorder is in great demand due to the rise of bilingualism worldwide and growing awareness of the communication needs in different populations (Garraffa et al., 2023). Phonological development in a group of bilingual speakers with Williams syndrome (WS) were investigated in a study on trajectories in late phonological development (Pérez et al.). Individual differences with a tendency for omissions, e.g., of final consonants, have been reported as specific to WS at all ages. The study brings together evidence for an appropriate phonological assessment and treatment for people with WS across the lifespan.

In a scoping review on possible effects of bilingualism on cognition and behavior in speakers with attention deficit hyperactivity disorder (ADHD), Köder et al. reported a lack of research and no consistent evidence in support of the hypothesis that bilingualism can mitigate or act as a barrier in speakers with ADHD. Two main outcomes are reported in the review: first, there should not be any concern about individuals with ADHD learning additional languages; second, researchers should be cautious in investigating this topic, as studies are very diverse in the methodology and their scope.

## Section 2: language learning in a multilingual context

Learning new words is a common experience for a multilingual speaker that must develop two vocabularies. By studying the acquisition of cognates in trilingual speakers, Xue et al. reported a facilitatory effect from the first language (L1) on novel word learning and a strong interference of the second language (L2), suggesting that the multilingual experience can lead to different outcomes in word learning.

Pragmatic abilities in the speech of L2 speakers can be affected by contextual variables which may affect their oral production. By investigating oral speech act production in Chinese English as a foreign language (EFL) learners, Huang and Lu showed that some speech acts are more difficult than others and contextual variables may have distinct effects on different speech acts. These findings have important implications for the L2 classroom and can inform pedagogical approaches to teaching L2 pragmatics.

Focusing on reading in bilingual children and adults, Friesen et al. examined reading comprehension and reading strategies use. Bilingual adults reach comparable reading comprehension performance to monolingual children despite their lower vocabulary by using several strategies, e.g., inferencing. The study puts an emphasis on the role of effective strategies for reading comprehension in addition to language instruction.

In a study on narrative abilities in bilingual children's speakers of Urdu-Cantonese, Chan et al. investigated the relationship between macrostructure and microstructure between languages. Although the number of different words was consistently a positive predictor of the macrostructure's components in both languages, other microstructure measures showed mixed results, suggesting that the relationship between macrostructure and specific microstructural abilities can manifest similarly and differently between the L1 and the L2.

The grammatical skills of Dutch children with 22q11.2 Deletion Syndrome were investigated by Boerma et al. in comparison with children with DLD using spontaneous speech and standardized assessments, showing that although children with 22q11.2 had more deficits in their receptive language skills compared to children with DLD, there was an overlap between the two groups in their expressive language skills. This suggests that there are multiple routes to deficits in grammatical skills despite differences in etiology.

Farsi-English Heritage language speakers were investigated in the study by Komeili et al. that profiled vocabulary, morphosyntax, and narrative microstructure skills in both languages. The study demonstrated that on the vocabulary and narrative tasks participants were more dominant in English than in Farsi, while on grammar there were no significant differences between the two languages, supporting the importance of measuring language across multiple domains in studies of bilingual children.

## Author contributions

MG: Writing—original draft, Writing—review and editing. MTG: Writing—review and editing. TM: Writing—original draft, Writing—review and editing. GM: Writing—review and editing.
